# Network analysis of trait aggression among community youths and juvenile offenders

**DOI:** 10.1186/s40359-024-01872-w

**Published:** 2024-07-10

**Authors:** Chen Chen, Chenglong Li, Shienyu Fei, Wei Chen

**Affiliations:** 1 School of Psychology, Guizhou Normal University, Guiyang, China; 2 Inner Mongolia Student Bullying Prevention Research Center, Tongliao, China

**Keywords:** Trait aggression, The Buss-Perry aggression questionnaire, Network analysis, Juvenile offenders

## Abstract

**Objective:**

The mainstream view in trait aggression research has regarded the structure as representing the latent cause of the cognitions, emotions, and behaviors that supposedly reflect its nature. Under network perspective, trait aggression is not a latent cause of its features but a dynamic system of interacting elements. The current study uses network theory to explain the structure of relationships between trait aggression features in juvenile offenders and their peers.

**Methods:**

Network analysis was applied to investigate the dynamic system of trait aggression operationalized by the Buss-Perry Aggression Questionnaire in a sample of community youths (Mage = 17.46, *N* = 715) and juvenile offenders (Mage = 18.36, *N* = 834).

**Results:**

The facet level networks showed that anger is a particularly effective mechanism for activating all other traits. In addition, anger was more strongly associated with physical aggression and the overall network strength was greater in juvenile delinquency networks than in their peers. The item level networks revealed that A4 and A6 exhibited the highest predictability and strength centrality in both samples. Also, the Bayesian network indicated that these two items were positioned at the highest level in the model. There are similarities and differences between juvenile delinquents and community adolescents in trait aggression.

**Conclusion:**

Trait aggression was primarily activated by difficulty controlling one’s temper and feeling like a powder keg.

**Supplementary Information:**

The online version contains supplementary material available at 10.1186/s40359-024-01872-w.

## Introduction

It is not unexpected that certain groups, such as juvenile delinquents, may display higher levels of aggression compared to their peers [1]. As an inherent trait of human beings, the scientific interest in aggression has increased and attracted attention in many fields, especially regarding its nature [2]. To date, aggression has been defined in several different ways and applied to widely divergent forms of behavior. Social psychologists treat aggression as any behavior that is intended to harm another individual against their will [3]. Personality psychologists define aggression as a multidimensional construct, that is, a stable and persistent style of cognitive and emotional components (such as hostility and anger), and behavior components (such as verbal and physical aggression) that can be measured on a continuum of individual differences [4, 5]. In general, trait aggression can predict an individual’s level of aggressive behavior, which means that people with high trait aggression show more aggressive behavior than other individuals [6].

Some researchers have attempted to integrate the previous theories of aggression in order to establish a unified theoretical framework [7]. Among them, the General Aggression Model [GAM; 3, 8] is a more comprehensive theoretical model of aggression that explains the causes and consequences of aggressive behavior [2]. It emphasizes three main elements of the interaction between a person and a situation, namely inputs, routes and outcomes. Personal factors such as trait aggression influence an individual’s internal state (cognitive, affective and physiological arousal) through a series of interrelated routes. The cognitive route includes scripts related to hostility. The affective route includes an increase in state anger or general negative effects. It also includes the activation of actions related to aggression. The tendency to awaken from unrelated sources is referred to as arousal. All these routes lead to the decision-making process, which leads to aggressive behavior [8].

To date, several reliable tools have been developed to measure people’s level of aggression [5, 9, 10]. Many of these instruments conceptualize aggression as a trait-like construct, indicating a person’s overall propensity for aggressiveness in daily life. Among them, the Buss-Perry Aggression Questionnaire (BPAQ) stands out as a widely recognized and validated psychometric instrument in trait aggression research [4]. It consists of 29 items divided into four categories: hostility, anger, physical aggression, and verbal aggression. Hostility encompasses cognitions of antipathy and injustice, reflecting the cognitive aspect, while anger signifies physiological arousal and embodies the emotional dimension. Physical aggression and verbal aggression, on the other hand, are indicative of aggressive behavioral characteristics. Evidently, the four-factor model appears to align with the conceptual framework of GAM and is considered the gold standard for measuring trait aggression.

In terms of scale measurement, various studies utilizing factor analytic methods have consistently determined a four-factor solution as the most suitable latent model for the BPAQ’s items across many diverse groups, languages, cultures, and contexts [11–14]. Since the BPAQ subscale and total scores have been shown to predict aggressive inclinations within the general population, numerous researchers have applied this measure to examine variations in aggressiveness levels among offender and non-offender groups [15–17].This four-factor approach has established a foundation for exploring aggression from a multidimensional perspective, facilitating investigations into the connections between aggression’s distinct elements and other psychological constructs like personality [18, 19], as well as core emotion regulation processes [8].

This scale enhances understanding of the various aspects of trait aggression. However, the conventional perspective in trait aggression research predominantly focuses on latent variable models. These models interpret the role of trait aggression as common cause behind the emergence of aggressive cognitions, emotions, and behaviors. Many studies have reported the latent variable method, which considers all items to be interchangeable and can aggregate the scores of all items when calculating subscales or total scores [20]. Latent variable models operate under the assumption of conditional independence, positing that observable indicators are independent from one another. The assumption of local independence contradicts the notion that cognitive, affective, and behavioral components—or items—are directly interconnected for causal, homeostatic, or logical reasons, thus clashing with the prevailing latent trait approach to personality dimensions and their corresponding items [20].

In recent years, a variety of research fields have started from the perspective of the network, which is consistent with the increasing interest in system complexity modeling and the improvement of computing power [21]. Psychology has also begun to embrace the network method to analyze psychological constructs [22]. From the perspective of the network, researchers attribute the emergence and covariation between the unique constituent features of the psychological structure of interest to the direct interaction between the elements themselves [23–25]. In this way, the network method is different from the latent variable model, it treats the psychological structure of interest (i.e., aggression) as caused by the interaction of its components [20, 23].

The network theory of psychological constructs proposed by Cramer et al. [26], has been applied to different personality traits and mental disorders [27], such as conscientiousness [28], alexithymia [29], trait anxiety [25], trait rumination [30], callous-unemotional traits [31] and so on. However, as far as we know, few studies have explored the network structure of trait aggression. One study utilized the Aberrant Behavior Checklist (ABC) as a measurement tool to analyze the network structure of irritability and aggression in individuals with autism spectrum disorder (ASD) [32, 33]. It found that irritability symptoms play a more pivotal role than aggression symptoms within the network. Given that ABC is not a tool specifically designed to measure trait aggression, we conducted network analyses of the items comprising Buss-Perry aggression questionnaire. In a separate study, researchers examined the integration of trait aggression into the broader frameworks of personality, revealing that trait aggression is mainly a subordinate facet of agreeableness [34]. While these findings enrich our comprehension of trait aggression within the Big Five personality framework, they overlook the interplay between the four components of trait aggression. Additionally, the study’s reliance on a community-based adult sample limits its generalizability to adolescent populations.

The purpose of current study is to investigate the network structure of trait aggression using community youths and juvenile offenders in China. First, we computed a graphical Gaussian model (GGM) model, which can directly reflect the correlation between nodes [35]. Relatedly, we also measured the significance of each node in generating the network structure by computing centrality metrics [36]. Second, we used network comparison test to compare the differences of the network structures of the two samples. Third, the directed acyclic graph (DAG) that best depicts the conditional independence links among aggression characteristics was computed using a Bayesian technique as a last step in our investigation into the causal structure of the aggressiveness network [37]. This technique provides theories about possibly causative relationships by not only identifying direct correlations between pairs of features but also estimating the direction of the correlation.

## Method

### Participants

#### Community sample

The Sample included 864 participants (35.8% male) from a middle school and a technical secondary school in Guizhou Province, China. The participants were 14 to 24 years old (M = 17.46, SD = 2.37). Most students were ethnic minorities (56.6%). 90% of the students do not come from one-child families. The students have signed the informed consent form and completed the questionnaire under the guidance of the teacher. Upon completion of the questionnaire, participants received a modest reward.

#### Offender sample

We recruited 723 juvenile offenders in China. These participants were recruited from the juvenile correctional facility through educational activities. Participants were between 11 and 26 years old (M = 18.36, SD = 2.40). According to the suggestion [24, 25], participants with missing values were excluded (*n* = 8). The analyses were thus performed on the remaining 715 participants.

### Measures

#### Buss-Perry aggression questionnaire – chinese version

We used 25-items BPAQ-CV to measure trait aggression [38]. It includes four subscales, each assessing one of the four facets: (a) seven items measure the Physical aggression; (b) five items measure the Verbal aggression facet; (c) six items measure the Anger facet, and (d) seven items measure the Hostility facet. Participants rate each item on a 5-point Likert-type scale, ranging from 1 (extremely uncharacteristic of me) to 5 (extremely characteristic of me). For each facet, higher scores indicate higher aggression.

The BPAQ-CV and each of its facet-related subscales show extremely high reliability and validity. Estimates indicated excellent internal consistency in both the community samples (Cronbach’s α = 0.88, McDonald’s ω = 0.89) and the juvenile offenders (Cronbach’s α = 0.90, McDonald’s ω = 0.90).

### Network analysis

Data were examined using R-studio, a program based on R (version 4.2.2). Network estimation and visualization are handled by the packages *qgraph* [39] and *bootnet* [40], predictability is handled by *mgm* [41], network structure comparison is handled by *NetworkComparisonTest* [42], and directed acyclic graphs are handled by *bnlearn* [43].

#### Estimation of the graphical gaussian model

A network structure is composed of nodes and edges: a node represents an item from the BPAQ, and edges are connections between two items. A regularized partial correlation network was estimated based on the correlation matrix of four facets and 25 items. We present a graphical gaussian model (GGM) that was regularized through the graphical Least Absolute Shrinkage and Selection Operator algorithm, which has several advantages [35]. The most obvious advantage is that it eliminates false associations caused by the influence of other nodes in the network by estimating the regularized partial correlation between pairs of nodes. In addition, it reduces the trivial small associations to zero, resulting in a sparse network containing only the strongest edges. We did so via the R package *qgraph*, which automatically implements such a regularization along with model selection based on the Extended Bayesian Information Criterion (EBIC). The estimation procedure selected the network with the lowest lambda value (lambda being the tuning parameter for this procedure) from the 100 networks; in these situations, we followed the recommendation to set the tuning parameter to 0.001 [35]. A final model is chosen according to the lowest EBIC value, given a specific hyperparameter gamma γ, typically set to 0.5, which was shown to yield accurate network estimations [44, 45].

#### Node centrality

Strength and node predictability were calculated to represent the importance of each node in the network. Strength was the sum of a node’s connections and represented the relative importance of a node in a network [46]. Node predictability was calculated as the percentage of shared variance between a node and its neighbors in the network, which provided a measure of absolute importance of a node [47]. We used two-step Expected influence as an index of bridge centrality to investigate the degree of intercommunity influence [48].

#### Stability and accuracy

Two types of robustness analyses were performed using the emerging R-package *bootnet* [40]. Firstly, we examined the accuracy of the undirected network by bootstrapping the edge weights and constructing 95% confidence intervals (with 5,000 bootstrapped samples). A lower degree of overlap between these CIs indicates higher accuracy. Secondly, we assessed the stability of node centrality estimates by running a subsampling procedure (with 5,000 bootstrapped samples), where a certain proportion of participants was removed and the network was recalculated. If the centrality estimates of the resulting network, after excluding many samples, were highly correlated with the centrality order of the original network, it can be considered stable. Additionally, we calculated the centrality stability coefficient (CS-coefficient) as a reference measure for stability. A CS-coefficient greater than 0.25 indicates acceptable stability, while a value exceeding 0.5 denotes good stability.

#### Network comparison test

We used a permutation hypothesis test called network comparison test (NCT) to investigate the differences of network structure between community youths and juvenile offenders. The NCT evaluates three assumptions commonly associated with network analysis: (1) constant network structure, (2) constant edge strength, and (3) constant global strength. The first assumption pertains to the overall network structure, positing that it is identical across different groups. The second hypothesis, in contrast, focuses on the varying intensities of a specific edge within the network. The third assumption is that although the network structure may be different, the overall level of connectivity between the groups is equal.

#### Directed acyclic graph

In order to calculate and visualize Bayesian networks, we ran the hill-climbing algorithm provided by R package *bnlearn*. The bootstrap function optimizes Bayesian information Criteria (BIC) by adding, subtracting, and inverting structural aspects of the network. The first step determines whether there is an edge between two nodes. We then restart the process randomly, using various candidate edges to possibly connect different pairs of nodes, interfere with the system, and so on. As this iterative process unfolds, the algorithm identifies the structure of the network. To ensure the stability of the DAG, we conducted bootstrapping 5000 times and averaged them to obtain the final network. We retained the edges that displayed the same direction in at least 85% of bootstrap networks in the final network [37].

## Result

### Descriptive statistical analysis

The mean and standard deviation of each item are depicted in Table 1. The higher the score, the stronger the trend of the feature. Mean four factors and overall trait aggression in the community sample was lower than in the offender sample, effect size ranged from 0.34 to 0.72 (see Supplemental materials, Table S1).

**Table 1:** Summary of descriptive analysis in both samples

Item	Community sample (*N* = 864)		Offender sample (*N* = 715)	
	M	SD	M	SD
Physical Aggression	16.05	5.04	19.32	5.30
1. If I have to resort to violence to protect my rights, I will.	2.67	1.22	3.15	1.12
2. I have become so mad that I have broken things.	2.14	1.23	2.78	1.26
3. Once in a while I can’t control the urge to strike another person.	2.31	1.21	2.92	1.21
4. I have threatened people I know.				
5. Given enough provocation, I may hit another person.	2.11	1.18	2.43	1.15
6. I can think of no good reason for ever hitting a person.”				
7. If somebody hits me, I hit back.	3.05	1.19	3.20	1.18
8. There are people who pushed me so far that we came to blows.	2.05	1.11	2.51	1.08
9. I get into fights a little more than the average person.	1.71	1.02	2.34	1.15
Verbal Aggression	12.83	3.47	15.24	3.23
I. I tell my friends openly when I disagree with them.	2.94	1.11	3.33	1.02
2. I can’t help getting into arguments when people disagree with me.	2.74	1.08	3.12	1.10
3. When people annoy me, I may tell them what I think of them.	2.58	1.06	3.10	1.05
4. I often find myself disagreeing with people.	2.37	1.04	2.91	1.03
5. My friends say that I’m somewhat argumentative.	2.21	1.09	2.77	1.00
Anger	14.84	4.76	16.48	4.81
1. Some of my friends think I’m a hothead.	2.35	1.11	2.58	1.18
2. I am an even-tempered person.”				
3. I flare up quickly but get over it quickly.	3.05	1.24	3.19	1.14
4. I have trouble controlling my temper.	2.40	1.12	2.72	1.13
5. When frustrated, I let my irritation show.	2.52	1.11	2.69	1.11
6. I sometimes feel like a powder keg ready to explode.	2.31	1.19	2.64	1.13
7. Sometimes I fly off the handle for no good reason.	2.21	1.19	2.66	1.14
Hostility	17.91	5.06	20.28	4.56
1. When people are especially nice, I wonder what they want.	2.36	1.14	2.77	1.08
2. I wonder why sometimes I feel so bitter about things.	2.96	1.16	3.15	1.06
3. I am suspicious of overly friendly strangers.	2.71	1.19	3.01	1.07
4. I am sometimes eaten up with jealousy.	2.22	1.08	2.55	1.04
5. At times I feel I have gotten a raw deal out of life.	2.72	1.13	3.02	1.06
6. I sometimes feel that people are laughing at me behind my back.	2.45	1.16	2.90	1.07
7. Other people always seem to get the breaks.				
8. I know that “friends” talk about me behind my back.	2.49	1.15	2.87	1.08

### Facet level networks

#### Network estimation

We estimated facet level networks that included the four factors of trait aggression. The network has been accurately estimated, as the confidence intervals around the edge weights were moderate in both samples (see Figure S1). Stability estimates for edge weights (CS = 0.59) and node strength (CS = 0.51) were adequate for community sample. Offender sample network demonstrated excellent stability coefficients for edge wights and strength (CS > 0.75). The stability analysis figure can be found in the supplementary material.

#### Network edges

**Fig. 1:**
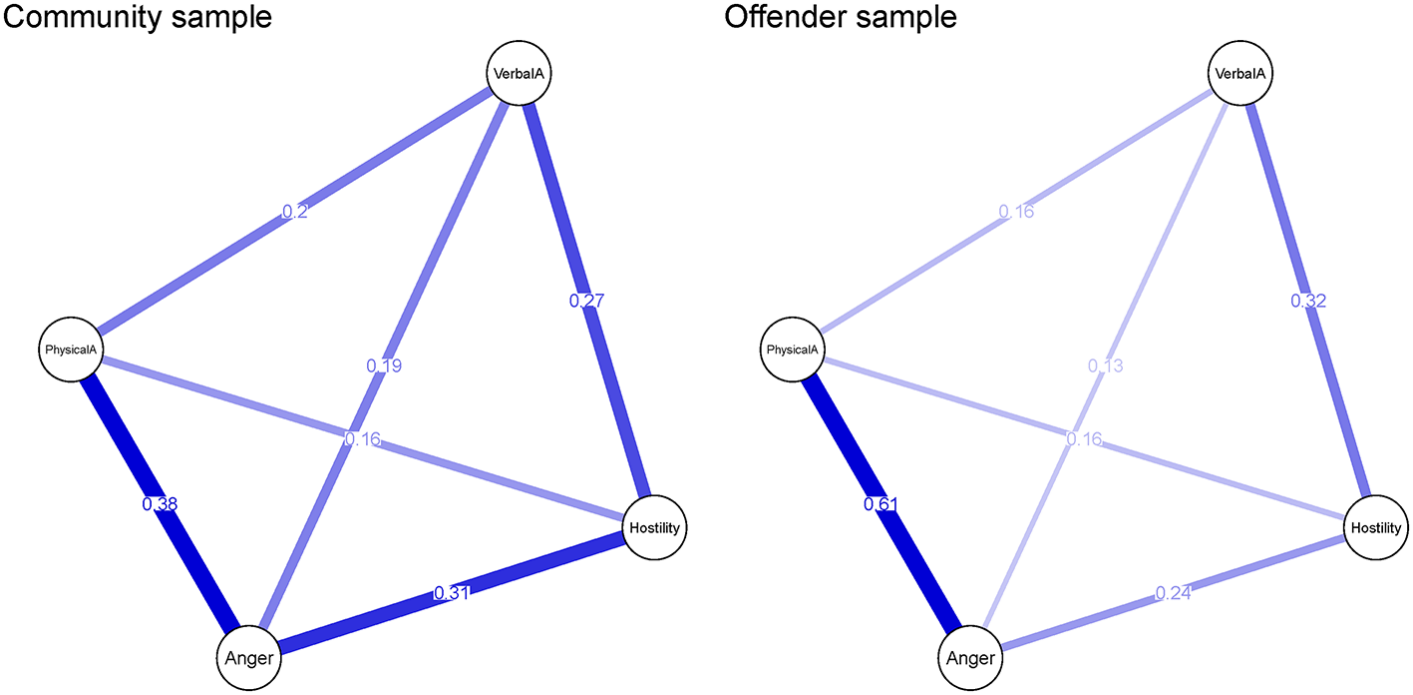
Facet level network structure of two samples. Blue edges represent positive associations. Edge width and depth of color indicate association strength

Figure 1 presents the estimated 4-facet graphical LASSO networks. Complete edge weight estimates for two networks are presented in Table S2 and Table S3. All of the connections between facets are positive, the edge wights ranged from small to moderate. The mean weight was 0.25 for the community sample and 0.27 for the offender sample. All networks revealed that the strongest edge wight between anger and physical aggression. In addition, anger and verbal aggression, as well as hostility and physical aggression had smaller edge weights in both networks.

#### Network centrality

Anger (0.88) was the most central node in the trait aggression network of community sample due to the edges it shared with the other traits. For offender, anger (0.98) and physical aggression (0.93) were highly central to network because of the strongest edge between these two traits. Verbal aggression had the lowest strength centrality in both samples (0.66 for community sample, 0.62 for offender sample).

#### Network comparison test

According to the NCT permutation test, there is statistical difference between the two networks in the global network structure (*M* = 0.225, *p* < 0.001). We performed a post-hoc test, and the results showed that there were significant differences in one edge (the edge between anger and physical aggression was larger in the offender sample network than in the community sample network). The two networks also differed significantly in global strength (1.51 for community sample, 1.63 for offender sample, *S* = 0.117, *p* < 0.001).

#### Directed acyclic graph

It was found that the direction probability of 12 arcs was exactly 0.5 in both samples through 5000 bootstrap sampling, indicating that the relationship between each trait was undirected, and the directed acyclic graph could not be generated at the facet level.

### Item level networks

#### Network estimation

To better understand which features are more important in trait aggression, we estimated two item level networks including 25 nodes. Networks demonstrated ideal stability coefficients for edge weights and centrality (CS > 0.5). The results of bootstrapped 95% confidence intervals for the edge weights and bootstrapped difference tests for edge weights and node strength are available in Supplementary materials (Figure S4 – S6).

#### Network edges

**Fig. 2:**
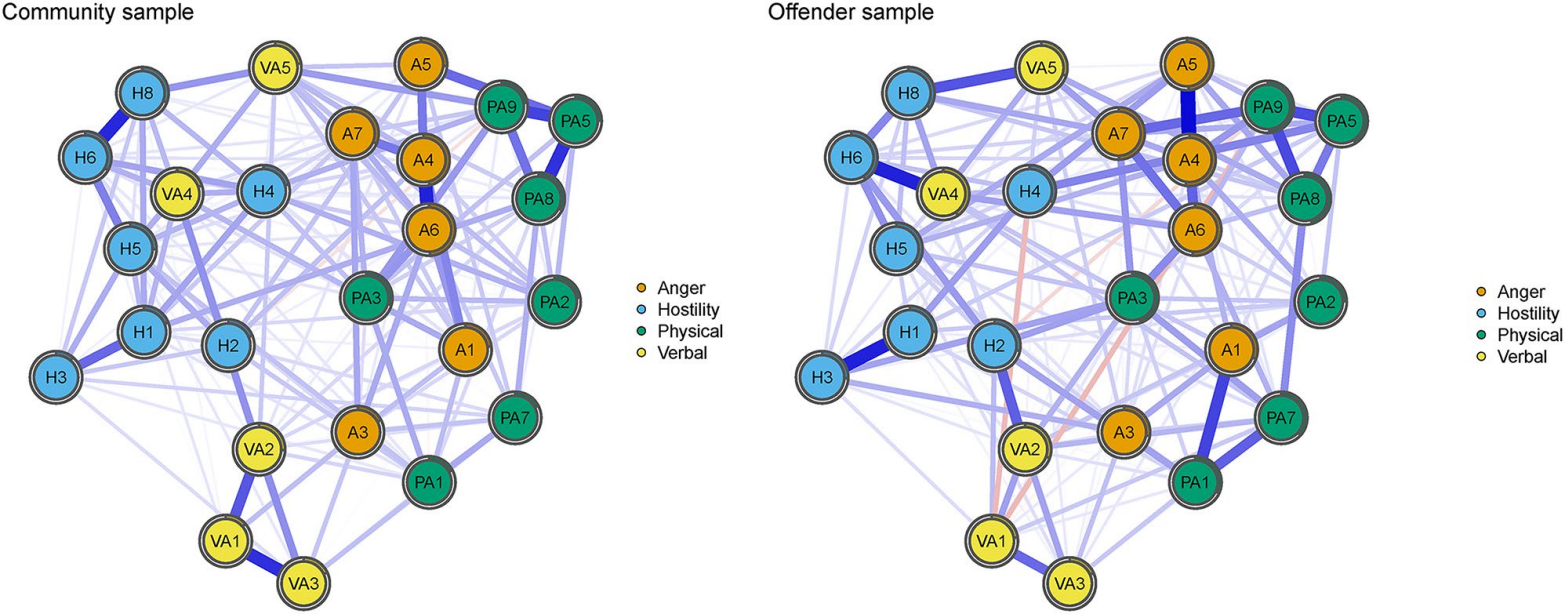
Item level network structure of two samples. The strength of the link is indicated by the line’s thickness. Positive regularized partial correlations are shown by blue lines, while negative regularized partial correlations are represented by red lines. Nodes with rings surrounding them show predictability

Figure 2 presents the estimated the 25-item graphical LASSO network. In the trait aggression network, most of the connections between items are positive, which indicates that the higher level of one item also indicates the unique differences of many other types of trait aggression. The number of non-zero edges of the community sample network was 169, and the average edge weight was 0.0346. Among them, there were five edge weights greater than 0.2 (A4–A6, H6–H8, VA1–VA3, PA5–PA8, PA5–PA9), and the strong edges all appeared among the items with the same traits. For the offender samples, network density was 0.55 (165 non-zero edges), and the mean weight was 0.0355, of which six edges had weights greater than 0.2 (A4–A5, H1–H3, VA4–H6, PA1–A1, PA8–PA9, PA5–PA9). All edge weights for both networks can be found in Supplemental Table S4 and Table S5.

#### Network centrality

The node predictability, node strength and bridge centrality of the two sample networks are shown in Table 2. ‘I have trouble controlling my temper’ (node A4) and ‘I sometimes feel like a powder keg ready to explode’ (node A6) have the highest node predictability and strength values for community sample network, which means they were the most central nodes. Similar results were presented in the offender sample network, with A4 and A6 having the highest predictability and relatively high node strength, and ‘Once in a while I can’t control the urge to strike another person’ (node PA3) having the greatest strength. In addition, the mean node predictability was 0.357 among juvenile offenders and 0.315 among community youths. Finally, ‘When frustrated, I let my irritation show’ (node VA5) in the community sample network and node PA3 in the offender sample network showed the greatest bridge centrality due to the connection with multiple node communities.

**Table 2:** Node centrality estimates from item level networks

	Predictability1	Predictability2	Strength1	Strength2	Bridge1	Bridge2
PA1	0.24	0.32	0.72	0.86	0.87	1.05
PA2	0.27	0.25	0.73	0.67	0.91	0.94
PA3	0.38	0.45	0.97	**1.14**	1.09	**1.54**
PA5	0.36	0.41	0.81	0.92	0.49	0.90
PA7	0.20	0.37	0.62	0.94	0.72	1.11
PA8	0.37	0.46	0.95	0.99	0.70	0.98
PA9	0.39	0.49	0.93	1.04	0.75	0.82
VA1	0.21	0.23	0.57	0.77	0.35	0.26
VA2	0.28	0.31	0.86	0.92	0.77	1.16
VA3	0.26	0.19	0.72	0.60	0.60	0.63
VA4	0.33	0.40	0.90	0.96	**1.28**	**1.52**
VA5	0.34	0.30	0.90	0.77	**1.41**	1.13
A1	0.32	0.29	0.81	0.79	0.86	1.03
A3	0.24	0.27	0.70	0.79	0.77	1.09
A4	**0.47**	**0.53**	**1.10**	**1.11**	0.92	1.07
A5	0.31	0.48	0.73	1.04	0.80	1.20
A6	**0.47**	**0.52**	**1.17**	**1.12**	1.07	1.34
A7	0.38	0.50	**0.97**	**1.06**	1.08	1.39
H1	0.29	0.25	0.86	0.72	0.72	0.62
H2	0.21	0.28	0.68	0.83	0.58	0.94
H3	0.18	0.26	0.55	0.72	0.35	0.64
H4	0.34	0.32	0.90	0.83	1.03	0.79
H5	0.34	0.34	0.97	0.82	0.88	0.97
H6	0.36	0.41	0.88	1.00	0.75	1.04
H8	0.37	0.34	0.96	0.80	0.78	0.93

*Note* 1 = Community sample, 2 = Offender sample, PA = Physical aggression, VA = Verbal aggression, A = Anger, H = Hostility

#### Network comparison test

The results indicated that there is statistical difference between the two networks in the global network structure (*M* = 0.170, *p* = 0.040), although this was not accompanied by differences in the global strength (10.49 for community sample, 11.12 for offender sample, *S* = 0.626, *p* = 0.124). The post-hoc test results showed that there were significant differences in 43 edges.

#### Directed acyclic graph

**Fig. 3:**
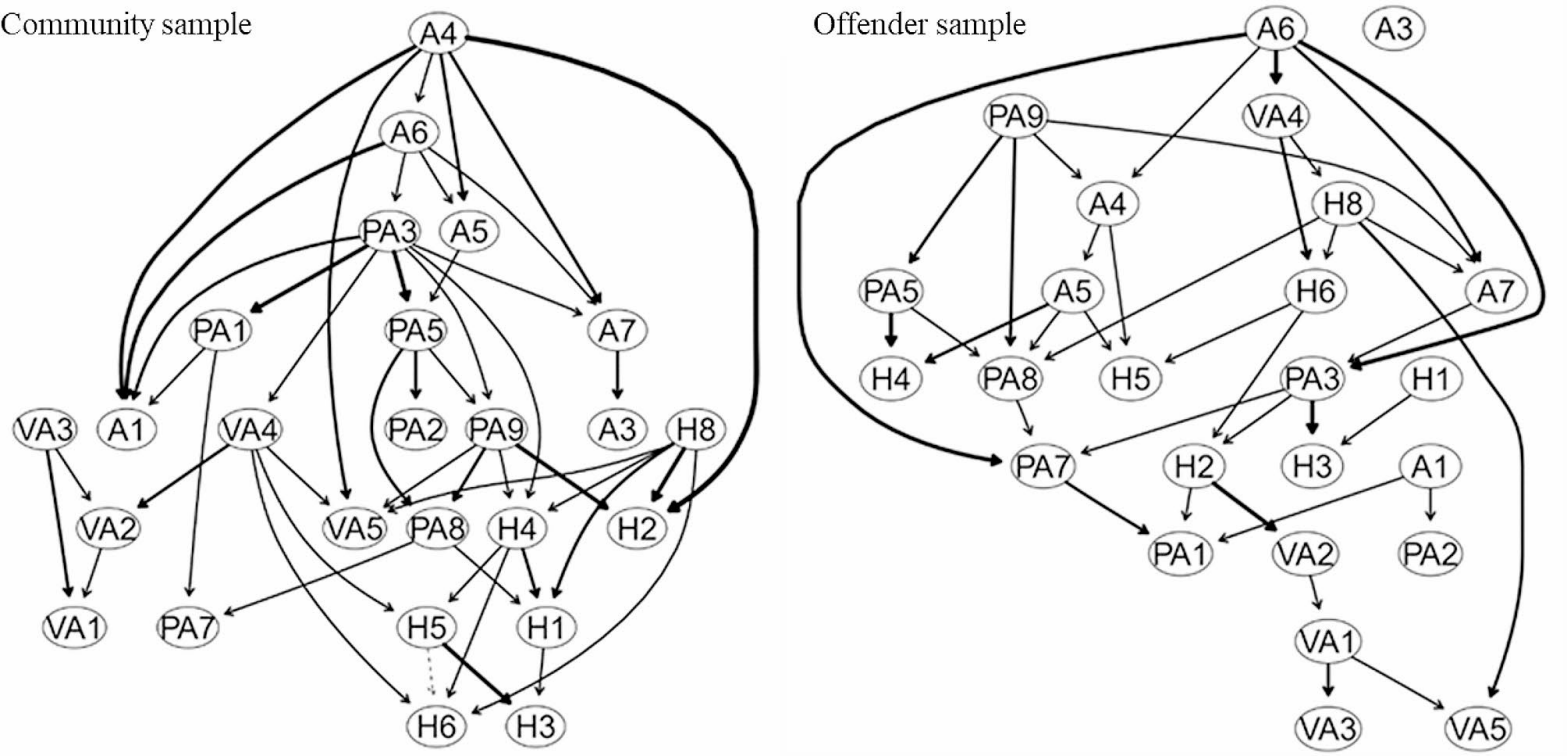
Directed acyclic graph (DAG) of 25 items trait aggression in community youth and juvenile offender. The direction of the presumptive causal links is shown by arrows. Edge thickness reflects the degree to which the expected direction of the edge points in the graph’s direction

Figure 3 depicts the DAG arising from the averaging of the 5000 bootstrapped networks and illustrates the association between items of trait aggression in two samples. Edge thickness signifies confidence that direction of prediction flows in the direction depicted in the graph. For community youths, ‘I have trouble controlling my temper’ (node A4) was positioned at the highest level in the Bayesian network, suggesting its causal priority. Specifically, difficulty controlling temper directly predicted other four anger items and also predicted being rated argumentative and bitter about things. In the DAG of juvenile offenders, ‘I sometimes feel like a powder keg ready to explode’ (node A6) was located at the vertices of the model. Most verbal aggression items are downstream features seemingly dependent on other aggression items in the network.

## Discussion

The four-factor conceptual model of trait aggression is one of the most prominent definitions of aggression in recent years [5]. In this study, we empirically examined the four-factor approach to trait aggression from a network perspective. To achieve this, we estimated the undirected network structure of aggressive traits among community youth and juvenile offenders to identify the central features of aggression and the interactions among its components. Additionally, a Bayesian hill-climbing algorithm facilitated the construction of a directed network that unveiled potential causal relationships among the components, thus contributing to the reproducibility and reliability of the research outcomes. The results demonstrated that anger is the most central factor and likely plays a critical role in initiating and perpetuating aggressive behavior. These insights improve our grasp of adolescent trait aggression and imply that clinicians ought to prioritize anger management in their behavioral interventions with adolescents.

We found that the network structures of trait aggression were similar across both samples, with items related to trait anger showing greater connectivity and a more prominent centrality index compared to other nodes. At the facet level, anger demonstrated elevated strength centrality values. These findings seem to be attributed to the influence of anger on trait aggression, given that all networks displayed relatively strong positive connections between anger and the remaining three facets of trait aggression. Further, the anger item nodes in item level networks were highly interconnected with the other three node communities. Again, the most salient feature of the DAG is its suggestion that trait anger, especially ‘I have trouble controlling my temper’ (node A4) and ‘I sometimes feel like a powder keg ready to explode’ (node A6), act as driving causal forces in the model. Previous research results also indicated that these two items have a higher factor load on latent traits and are retained in the modified 12-item BPAQ [49], which means that these two anger items may be key indicators that reflect trait aggression. Thus, it would seem that among the four dimensions of trait aggression, anger serves as its unifying factor. This conclusion seems to be in line with functional theories of anger as a driving force behind violent actions when other people do not regard our well-being enough [50]. The identification of trait anger as the primary driver of aggression should not be unexpected. Previous research has also shown a strong correlation between anger and the other three sub-traits during the initial development of the questionnaire [5]. Evidently, anger serves as a psychological link that connects instrumental and cognitive components. The network structure suggests that trait anger may indeed serve as the driving force of the entire aggression network system, which is consistent with the initial conceptualization of aggression within the GAM, which emphasizes the role of emotion processes as a critical link.

In addition to the commonalities, we also found differences between juvenile delinquents and community adolescents in trait aggression by using the network comparison test. The mean edge wights and global strength in offender samples are larger than in community youths, which means that the offender’s trait aggression network has high connectivity. Strong connectivity characterizes the state of greater susceptibility to trait aggression while weak connectivity reflects a resilient mental health state. This means that when a certain item of trait aggression is activated, it may spread to other characteristics through the network similar to the spread of a virus. By focusing therapeutic interventions on specific behaviors or thought patterns (nodes) that are more central or influential within the network of aggression, it might be possible to achieve a more substantial reduction in overall aggressive tendencies. Furthermore, the network structure of the two samples was significantly different at both levels, which may be mainly due to the relative position of physical aggression in the model. Since physical aggression was highly associated with anger, both were central to offender sample networks.

Our results also have implications for the measurement of trait aggression. Combining the results of all networks, we identify two items of anger as the core of trait aggression. In addition to the two items of anger, combined with the results of undirected network and Bayesian network, verbal aggression (node VA4:‘I often find myself disagreeing with people’ and node VA5: ‘When frustrated, I let my irritation show’), physical aggression (node PA3: ‘Once in a while I can’t control the urge to strike another person’ and node PA9: ‘I get into fights a little more than the average person’) and hostility (node H5: ‘At times I feel I have gotten a raw deal out of life’ and node H8: ‘I know that “friends” talk about me behind my back’) all have nodes that play an important role in the overall network structure. These items may not only be more representative of the corresponding trait, but also indispensable to reflect the overall trait aggression. Currently, some revised scales omit key items identified in our study [49, 51, 52], which may result in the loss of important information during the measurement process. Therefore, we suggest that the following surveying research can use the network method as an auxiliary means to retain the items.

The last key finding is a supplement to the existing literature, that is, the estimated network has good stability in both samples. The results about network analysis of trait aggression are highly dependent on stability and accuracy analysis. Stability is also crucial for the correct identification and clinical interpretation of targeted interventions. The present study, to our knowledge, was the first study using network approach in two different samples and the stability of the networks increases our confidence in the robustness and generalizability of the network structures.

The present study has several limitations that should be noted. First, one of our study’s main limitations is that the estimation of the GGM relies on cross-sectional data, thus excluding any strong inference regarding the potential causal relations between the features of aggression. Future research should apply longitudinal design from a development perspective to reveal the causal links between the items of trait aggression. Second, we only use a single scale to measure trait aggression as the four- factor model of this scale contains a comprehensive measure for trait aggression. In recent years, some researchers have questioned the four-factor model of trait aggression and proposed alternative measures [53, 54]. To deepen our understanding of trait aggression networks, future studies should also explore alternative measurement approaches. Moreover, as the present study relies solely on self-reported data, forthcoming inquiries should engage data from multiple informants and diverse samples to scrutinize the network structure of trait aggression more robustly. Lastly, given that past research has corroborated gender differences in aggressive behavior [56], it is crucial for subsequent studies to investigate the aggressive network characteristics in females, as this study was limited to examining only male offenders.

### Electronic supplementary material

Below is the link to the electronic supplementary material.

Supplementary Material 1

Supplementary Material 2

Supplementary Material 3

## Data Availability

Data is provided within the manuscript or supplementary information files.
