# Supplementary figures and images for: Network analysis of trait aggression among community youths and juvenile offenders
Source: BMC Psychol. 2024 Jul 10;12:387. doi: 10.1186/s40359-024-01872-w (PMC11238466; doi:10.1186/s40359-024-01872-w)

# Community sample

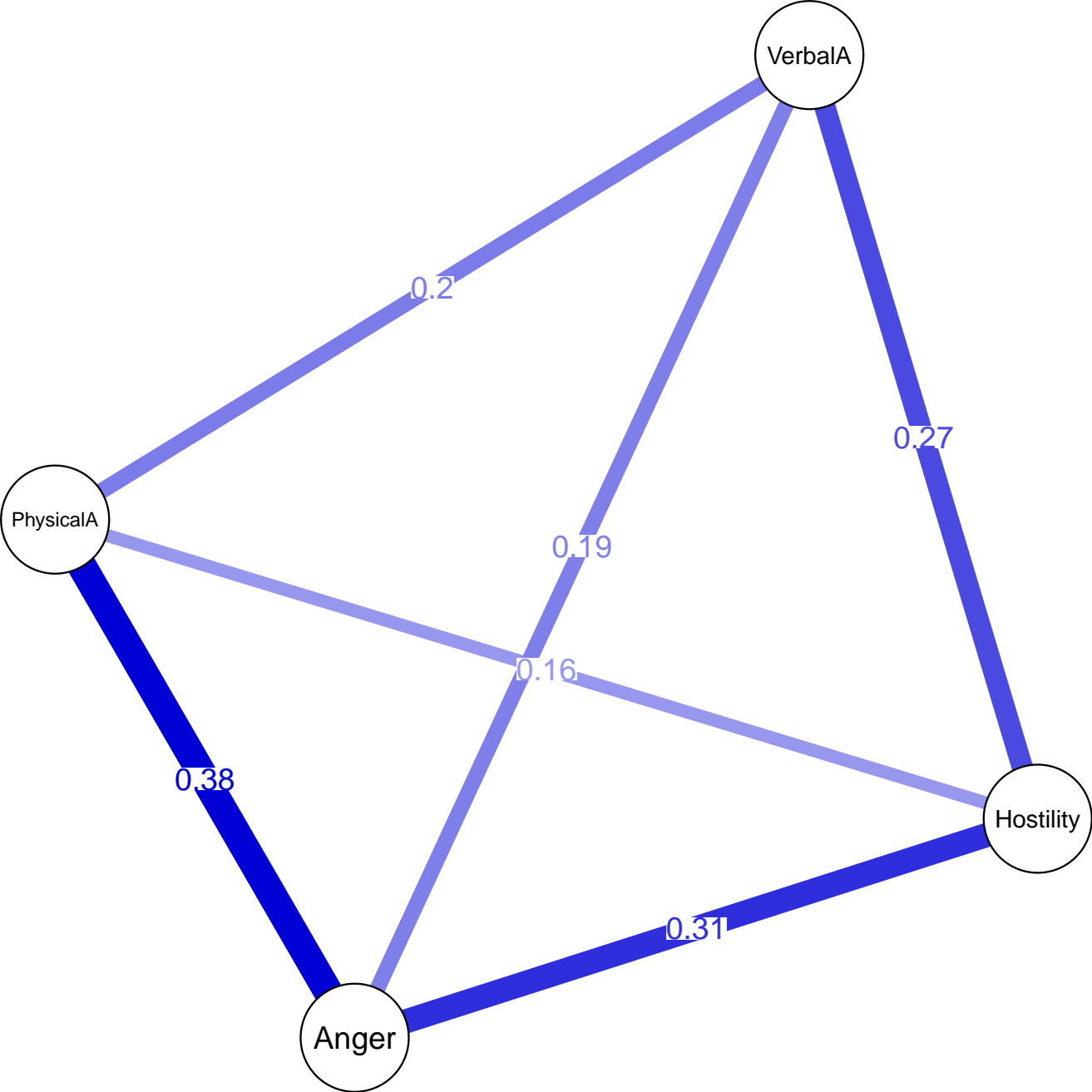

# Offender sample

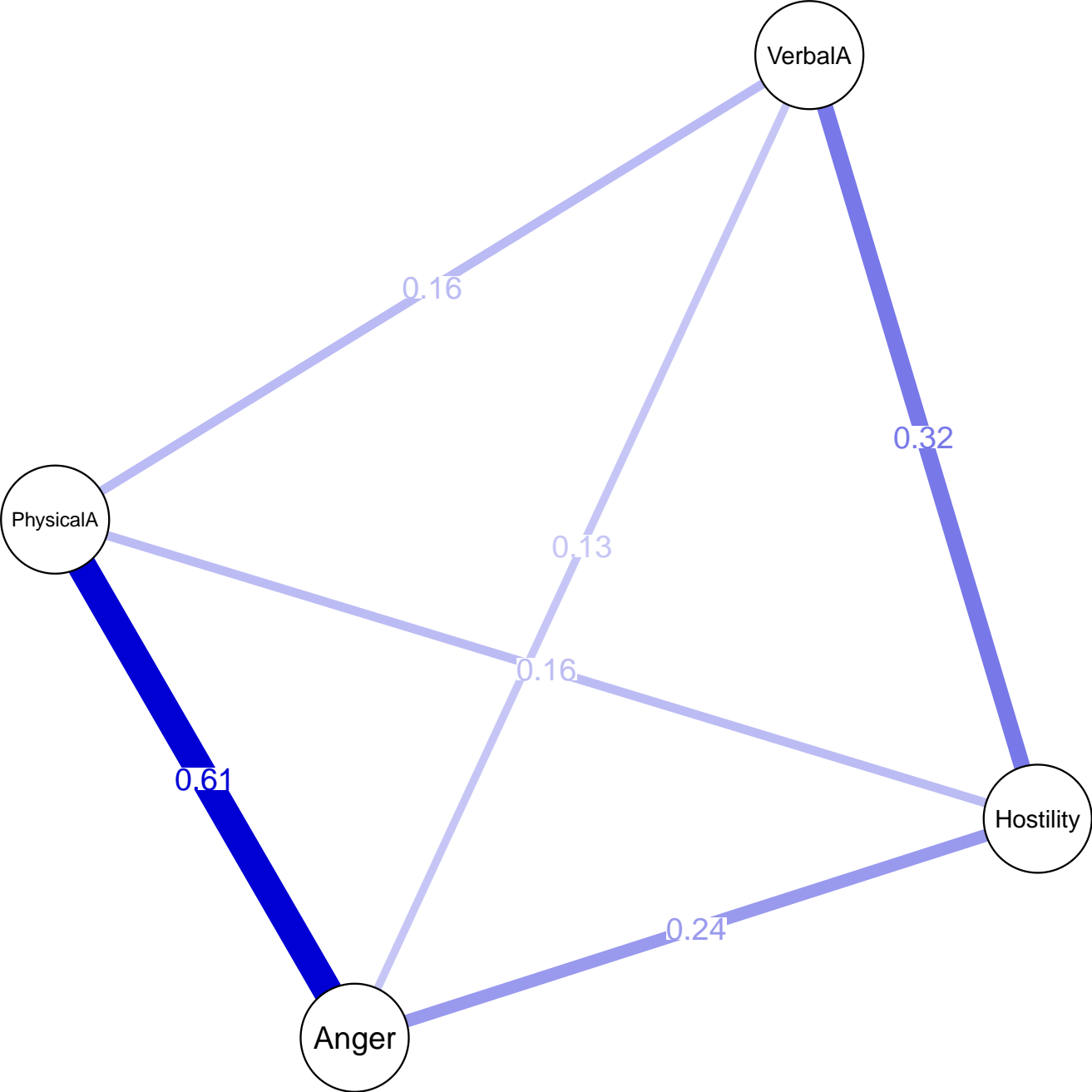

Supplement: Supplementary file 1 — Supplementary Material 1 [file 40359_2024_1872_MOESM1_ESM.pdf]

Community sample

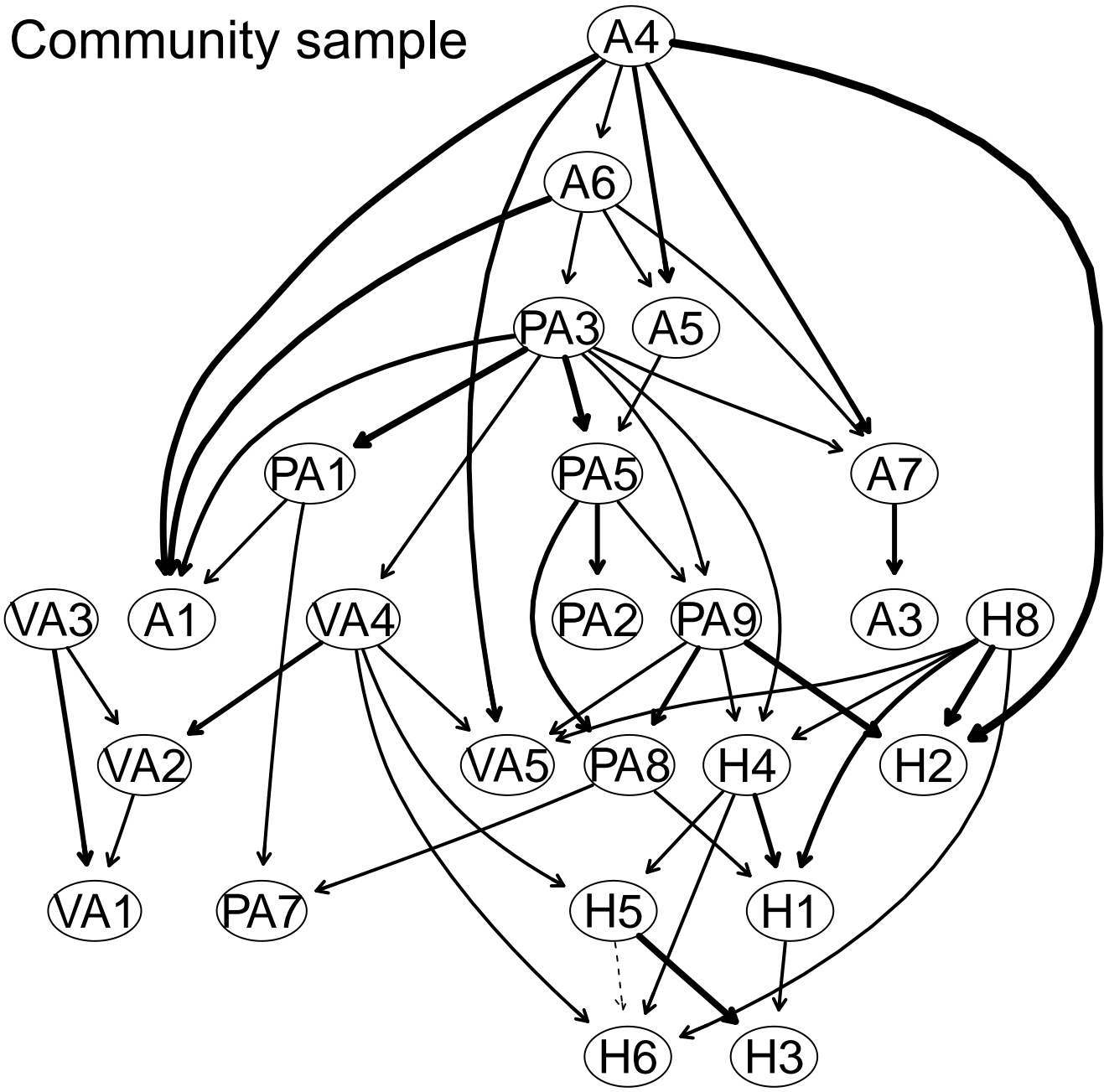

Offender sample

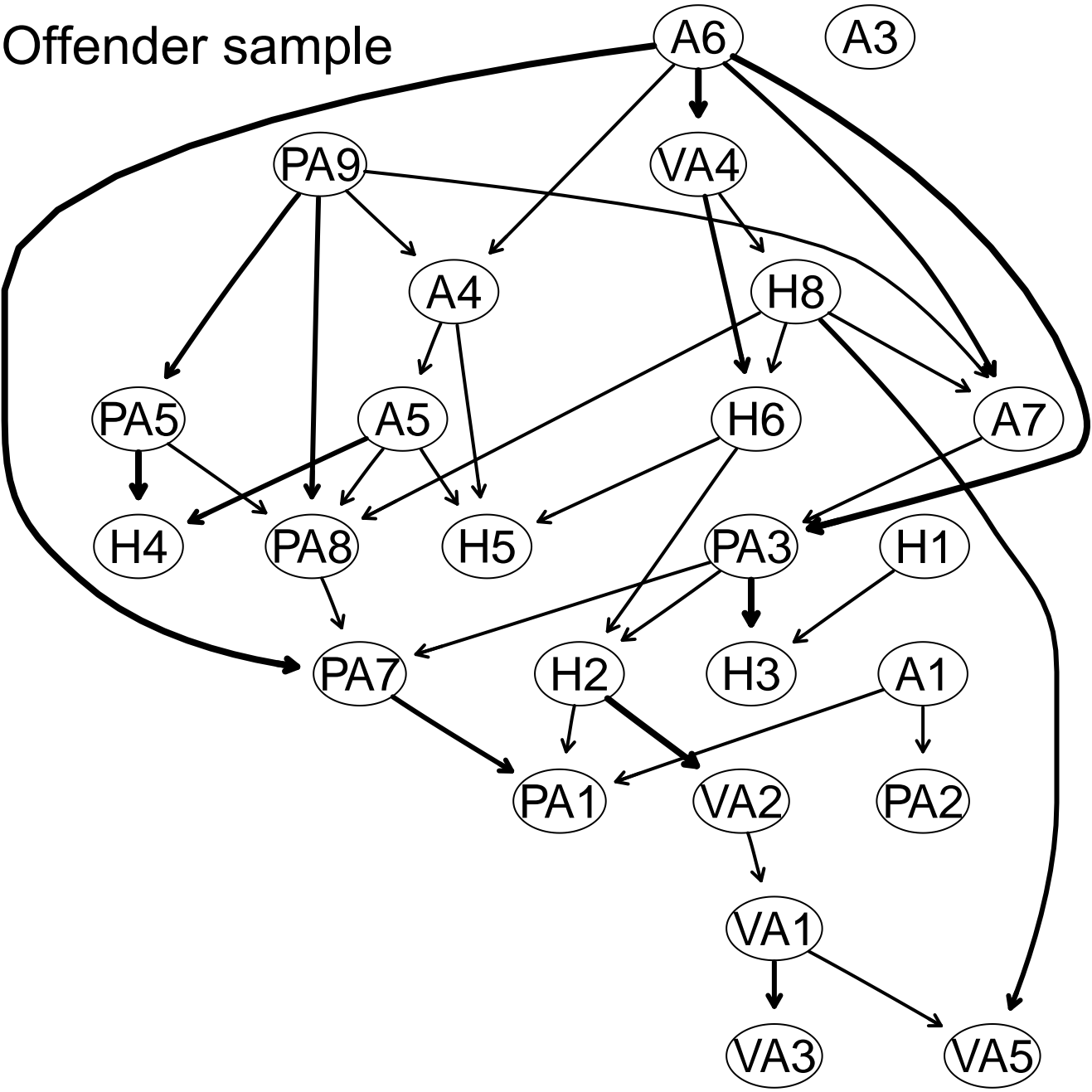

Supplement: Supplementary file 2 — Supplementary Material 2 [file 40359_2024_1872_MOESM2_ESM.pdf]

## Community sample

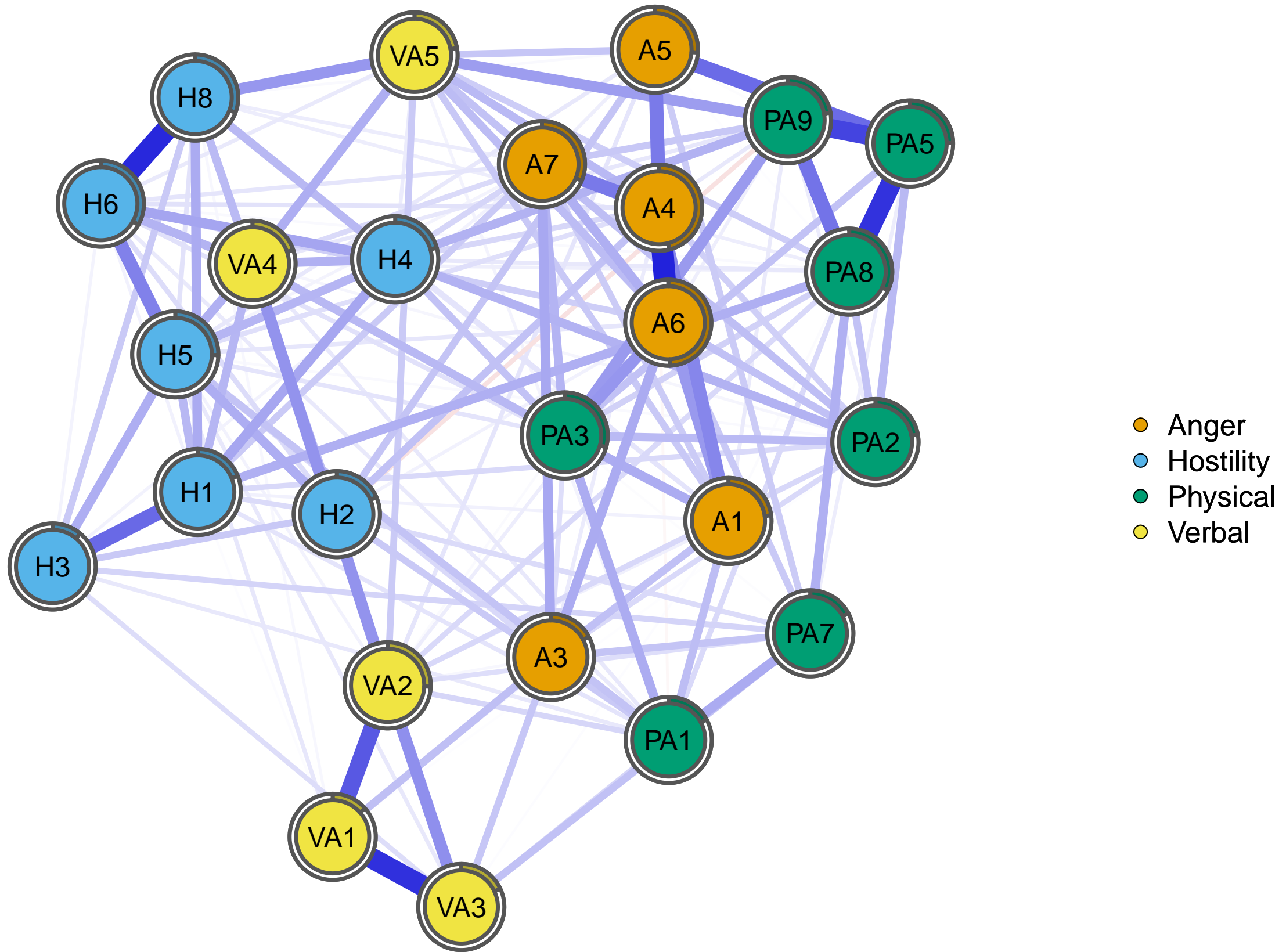

Offender sample

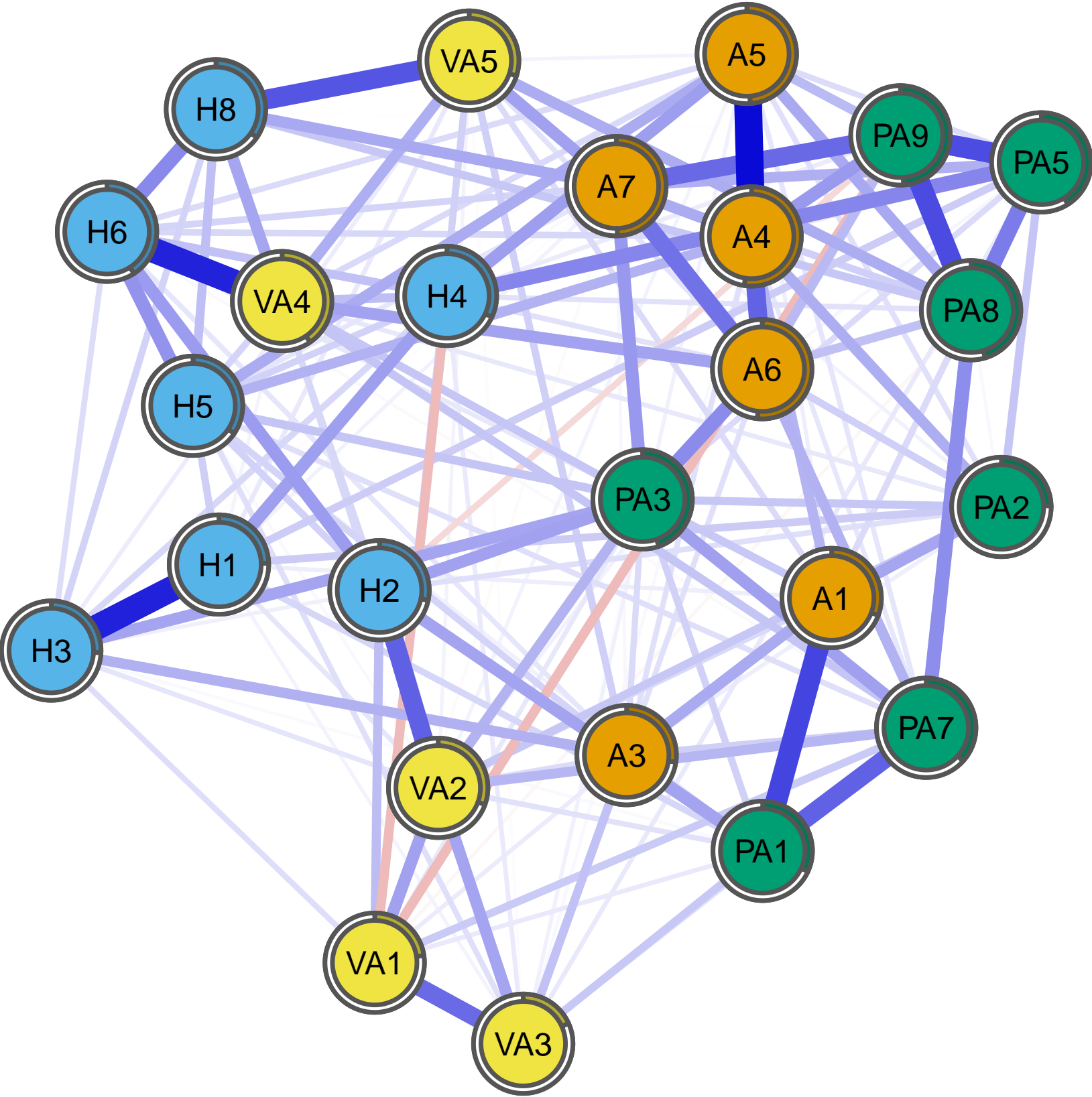

- Anger
- Hostility
- Physical
- Verbal

Supplement: Supplementary file 3 — Supplementary Material 3 [file 40359_2024_1872_MOESM3_ESM.pdf]
